# Targeting tumor multicellular aggregation through IGPR-1 inhibits colon cancer growth and improves chemotherapy

**DOI:** 10.1038/oncsis.2017.77

**Published:** 2017-09-18

**Authors:** N Woolf, B E Pearson, P A Bondzie, R D Meyer, M Lavaei, A C Belkina, V Chitalia, N Rahimi

**Affiliations:** 1 Department of Pathology, Boston University School of Medicine, Boston, MA, USA; 2 Flow Cytometry Core Facility, Department of Medicine, Boston University School of Medicine, Boston, MA, USA; 3 Department of Medicine, Boston University School of Medicine, Boston, MA, USA

## Abstract

Adhesion to extracellular matrix (ECM) is crucially important for survival of normal epithelial cells as detachment from ECM triggers specific apoptosis known as anoikis. As tumor cells lose the requirement for anchorage to ECM, they rely on cell–cell adhesion ‘multicellular aggregation’ for survival. Multicellular aggregation of tumor cells also significantly determines the sensitivity of tumor cells to the cytotoxic effects of chemotherapeutics. In this report, we demonstrate that expression of immunoglobulin containing and proline-rich receptor-1 (IGPR-1) is upregulated in human primary colon cancer. Our study demonstrates that IGPR-1 promotes tumor multicellular aggregation, and interfering with its adhesive function inhibits multicellular aggregation and, increases cell death. IGPR-1 supports colon carcinoma tumor xenograft growth in mouse, and inhibiting its activity by shRNA or blocking antibody inhibits tumor growth. More importantly, IGPR-1 regulates sensitivity of tumor cells to the chemotherapeutic agent, doxorubicin/adriamycin by a mechanism that involves doxorubicin-induced AKT activation and phosphorylation of IGPR-1 at Ser220. Our findings offer novel insight into IGPR-1's role in colorectal tumor growth, tumor chemosensitivity, and as a possible novel anti-cancer target.

## Introduction

To survive in tissue, epithelial cells must anchor to extracellular matrix (ECM), as detachment from it induces a specific programed cell death known as anoikis.^1^ Tumorigenic transformation due to genetic alterations allows tumor cells to survive and proliferate without the requirement of anchorage to ECM (that is, anchorage-independent growth).^2^ Resistance to anoikis plays a major role in tumor metastasis as tumor cells that survive after detachment from their primary location can travel through circulatory systems.^3^ Emerging evidence suggests that as tumor cells lose the requirement for anchorage dependency for growth and survival, they increasingly rely on their ability to adhere to each other (that is, multicellular aggregation) for survival.^4, 5^ Invasive tumors frequently invade stroma in large groups by the mechanism of collective cell migration.^6, 7^ Circulating tumors of colorectal, breast, and prostate cancer are often present in aggregates and not in a single cell.^8, 9, 10, 11^ Tumor cell aggregation also significantly influences the cells’ response to cytotoxic drugs, as tumor cells in a spheroid environment are more resistant to radiation and chemotherapeutic agents, a phenomenon originally coined multicellular resistance (MCR).^12, 13, 14, 15^ In this regard, *in vitro* multicellular spheroid cell culture conditions mimic the *in vivo* tumor microenvironment and interactive characteristics of solid tumors.^12, 16, 17^ Accumulating evidence on the role of cell–cell adhesion in tumor progression, and response to therapeutics suggests that tumor cell–cell interaction provides tumor cells an adaptive survival mechanism by which they overcome the need for anchorage dependency to ECM and evade the cytotoxic effects of chemotherapeutics.

Colorectal cancer (CRC) is one of the most common malignancies and one of the leading causes of cancer mortality.^18^ CRC can develop both from hereditary and non-hereditary sporadic mutations.^19, 20, 21^ Although inactivation of adenomatous polyposis coli (APC) and β-catenin are the most common and critical events in the initiation of CRC,^19, 22, 23, 24^ other genetic and cellular mechanisms by which tumor cells sense their microenvironment have profound importance in deriving the progression of malignancy and evasion from chemotherapy.^25, 26, 27, 28^ Understanding these key mechanisms in the face of drug resistance and non-responders to conventional therapies underlies any rational attempt to increase patients’ responses to treatments.

We recently identified immunoglobulin-containing and proline-rich receptor-1 (IGPR-1) as a novel member of the immunoglobulin (Ig) containing cell adhesion molecules (Ig-CAMs), which is broadly expressed in normal human epithelial and endothelial cell types.^29^ IGPR-1 is comprised of three major domains: extracellular, transmembrane and intracellular. The extracellular domain of IGPR-1 contains a single immunoglobulin domain followed by a single transmembrane domain and a proline-rich intracellular domain. The immunoglobulin-containing extracellular domain is required for IGPR-1 to mediate endothelial cell–cell interaction and barrier function.^29, 30^ The proline-rich intracellular domain of IGPR-1 is phosphorylated at multiple serine residues^30^ and associates with various Src homology 3 (SH3) domain-containing proteins, including SPIN90/WISH (SH3 protein interacting with Nck), potentially linking IGPR-1 to actin polymerization via N-WASP and Arp2/3 complex.^29^ In addition to its adhesive function, IGPR-1 binds to HHLA2, a member of the B7 family of costimulatory molecules involved in the activation and downregulation of T lymphocytes.^31^ In the present study, we have demonstrated that IGPR-1 is upregulated in colorectal cancer and provide evidence that it promotes multicellular aggregation in tumor cells, increases tumor growth *in vivo* and *in vitro*, and increases the resistance of tumor cells to doxorubicin.

## Results

### IGPR-1 expression is upregulated in human primary colorectal tumors

**Figure 1:**
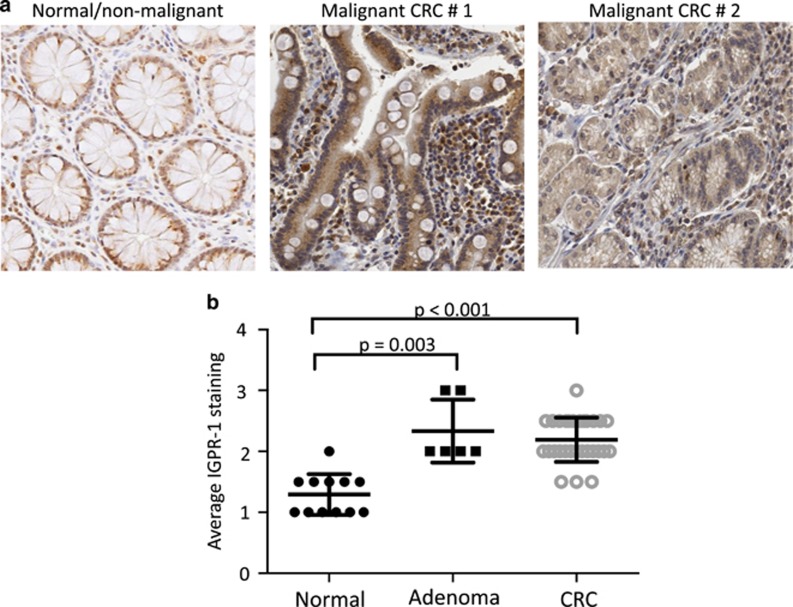
IGPR-1 expression is upregulated in human colorectal cancer. Human paraffin-fixed CRC biopsy samples (29 cases) from patients treated at Boston University Medical campus were stained with a polyclonal anti-IGPR-1 antibody. A representative IGPR-1 expression in normal and malignant CRC tumors (**a**). The graph compares the average staining of IGPR-1 in normal, dysplastic adenoma and malignant/invasive CRC tumors (**b**).

To investigate the potential role of IGPR-1 in human colorectal cancer (CRC), we examined expression of IGPR-1 in CRC by staining CRC biopsies of patients treated at Boston University School of Medicine using a pre-validated IGPR-1 antibody.^29, 30^ The cohort consisted of 29 human colorectal cancer specimens (19 cases of well or moderately differentiated CRC, 3 cases of poorly or undifferentiated CRC, and 7 cases of mucinous CRC), and 6 patients with non-neoplastic tubular adenoma were compared to 12 patients in whom normal colonic tissue was adjacent to CRC. Gastrointestinal surgical pathologists quantified the expression of IGPR-1 using a semi-quantitative scale (low to high expression corresponding to 1+ to 3+ staining; Figure 1a). The data revealed an increase in IGPR-1 level in both the adenoma and CRC compared to normal tissue (Mean±s.d. for normal tissue was 1.29±0.33; adenoma 2.33±0.51 and CRC 2.19±0.369; Figure 1b). Compared to the normal tissue, IGPR-1 levels were significantly increased in adenoma (Mann–Whitney *U*-test *P*=0.003) and CRC (*P*<0.001) indicating that the levels of IGPR-1 increased in early stages of CRC. The observation suggests that expression of IGPR-1 could have a role in the tumorigenic properties of CRC tumor cells.

### Targeting IGPR-1 in CRC inhibits tumor growth

**Figure 2:**
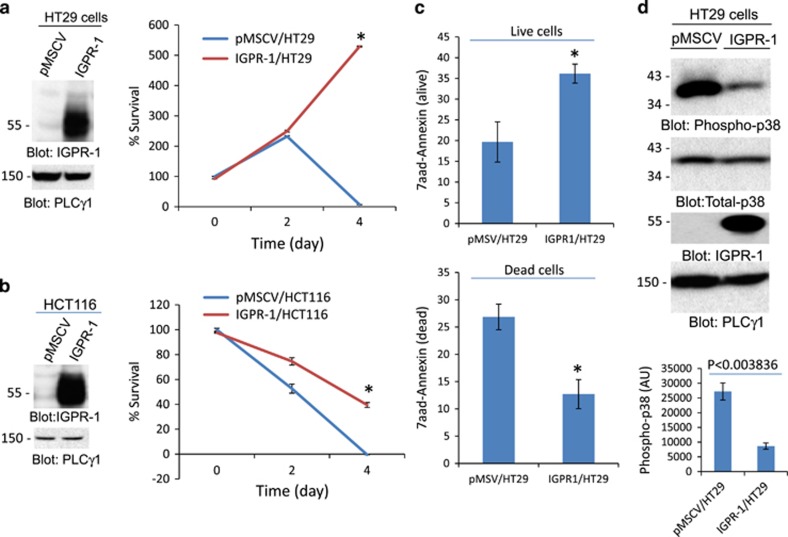
IGPR-1 promotes HT29 and HCT116 cells survival in suspension. Ectopic expression of IGPR-1 in HT29 and HCT116 cells is shown (**a**, **b**). HT29 and HCT116 cells expressing empty vector (pMSCV) or IGPR-1 were seeded in non-adherent 24-well plates (5 × 10^4^/well) in 10%FBS in quadruple wells/group. Cell viability was determined by MTT assay at day 0, 2 and 4 (**a**, **b**). HT29 cells expressing empty vector or IGPR-1 were seeded in non-adherent six-well plates and survival of cells was measured by flow cytometry analysis by staining of cells with 7AAD-Annexin V. Live cells were defined as calcein violet+, 7AAD−, Annexin V−, and apoptotic/dying cells were defined as Annexin V+ (**c**). HT29 cells expressing empty vector or IGPR-1 were seeded in non-adherent 24-well plates for 24 h. Cells were lysed and whole-cell lysates were blotted for phospho-p38, total p38, IGPR-1 or for PLCγ1 as a protein loading control (**d**).

To investigate a possible functional role of IGPR-1 in CRC tumors, we ectopically expressed IGPR-1 in human colorectal adenocarcinoma cell line, HT29 and human colorectal carcinoma cell line, HCT116 cells (Figures 2a and b). IGPR-1 is endogenously expressed at a low level in both HT29 and HCT116 cells (Figures 2a and b). To examine the effect of overexpression of IGPR-1 in HT29 and HCT116 cells, we measured the viability of tumor cells expressing empty vector or IGPR-1 in a tumorsphere assay (that is, non-adherent/suspension condition). The viability of cells was determined by MTT (3-(4,5-dimethylthiazol-2-yl)-2,5-diphenyltetrazolium bromide) assay, which measures metabolic activity of live cells. Tumorsphere assay is considered to mimic the *in vivo* tumor architecture more closely than the monolayer cell culture system.^32, 33^ IGPR-1 increased survival of both HT29 and HCT116 cells in suspension condition (Figures 2a and b). The prosurvival effect of of IGPR-1 in HT29 cells was significantly higher than its effect in HCT116 cells (Figures 2a and b). 7AAD-Annexin V staining further confirmed the prosurvival effect of IGPR-1 in HT29 cells in suspension. HT29 cells expressing IGPR-1 showed significantly higher cell survival and reduced apoptosis compared to HT29 cells expressing empty vector (Figure 2c). Intriguingly, IGPR-1 had no noticeable prosurvival effect on HT29 and HCT116 cells in adherent 2D cell culture condition (Supplementary Figure 1). The observation indicated that the ectopic expression of IGPR-1 in HCT116 and HT29 cells protects tumor cells from the suspension-induced apoptosis.

The extracellular domain of IGPR-1, through homophilic trans-dimerization, mediates cell–cell adhesion.^29, 30^ Therefore, we tested whether the extracellular domain of IGPR-1 is required for its prosurvival function. Deletion of the extracellular domain of IGPR-1 (ΔN-IGPR-1) significantly eliminated its prosurvival effect in HT29 cells in suspension condition (Supplementary Figure 2B). However, ΔN-IGPR-1 retained a baseline prosurvival activity (Supplementary Figure 2B), indicating that the cytoplasmic domain of IGPR-1 is capable of some prosurvival signaling in the absence of extracellular domain-mediated dimerization. To further elucidate the role of extracellular domain in IGPR-1 function, we generated a chimeric IGPR-1 by replacing the extracellular domain of IGPR-1 with the human colony stimulating growth factor-1 receptor (CSF-1R), hereafter called cIGPR-1, and expressed it in HT29 cells (Supplementary Figure 2C). This strategy allowed us to examine the prosurvival effect of IGPR-1 in an inducible manner by stimulating cells with CSF-1R ligand, CSF-1. Stimulation of HT29 cells expressing cIGPR-1 with CSF-1 promoted survival of HT29 cells in the tumorsphere assay (Supplementary Figure 2D), and the prosurvival effect of cIGPR-1 in the presence of CSF-1 was strikingly similar to the effect of expression of wild-type IGPR-1 in HT29 cells (Figure 2b).

Loss of cell adhesion to ECM triggers activation of stress-induced pro-apoptotic p38 MAPK.^34, 35^ Since IGPR-1 expression in HCT116 and HT29 cells promoted tumor cell survival in the absence of adhesion to ECM, we hypothesized that the ability of IGPR-1 to promote survival of tumor cells in tumorsphere conditions is mediated by inhibition of p38. Analysis of phosphorylation of p38 in HT29 cells showed that in HT29 cells expressing IGPR-1, phosphorylation of p38 was significantly inhibited (Figure 2d), suggesting that the prosurvival effect of IGPR-1 in CRC tumor cells in the absence of adhesion to ECM is mediated by reducing activity of the stress-induced p38. In support of this hypothesis, treatment of HT29 cells with p38 inhibitor, SB203580 attenuated apoptosis of HT29 cells in the tumorsphere assay (Supplementary Figure 3A), whereas treatment of HT29 cells expressing IGPR-1 with SB203580 enhanced the prosurvival effect of IGPR-1 (Supplementary Figure 3B).

**Figure 3:**
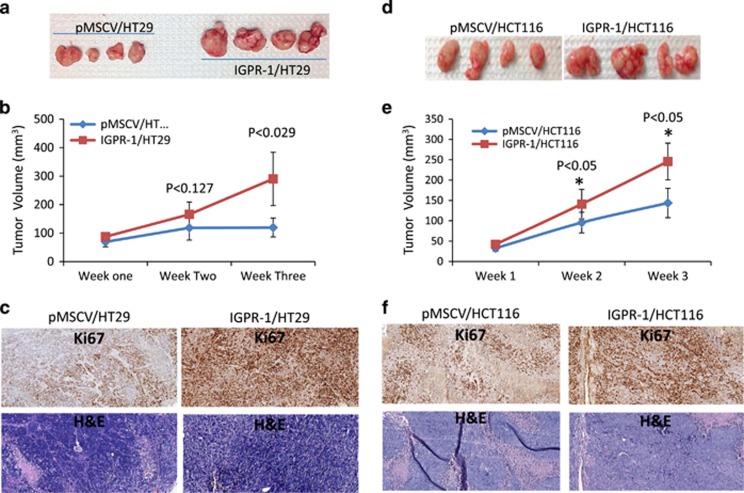
IGPR-1 supports CRC tumor cell growth in mouse xenograft. HT29 and HCT116 cells either expressing empty vector (pMSCV) or IGPR-1 were injected into nude mice (4 mice per group). The growth of tumors were measured every week up to three weeks and the average tumor volume is shown. At day 21, animals were sacrificed, tumors were removed and pictures were taken (**a**, **b**, **d**, **e**). Tumor tissues were fixed and stained with ki67, an *in vivo* proliferation marker, or H&E (**c**, **f**).

Considering the pronounced prosurvival effect of IGPR-1 in HT29 and HCT116 cells in the tumorsphere assay, we also examined whether expression of IGPR-1 in HT29 and HCT116 cells promotes *in vivo* tumor growth in an athymic mouse tumor xenograft model. The results showed that HT29 and HCT116 cells expressing IGPR-1 grew tumor significantly larger than the control tumor cells expressing empty vector (Figures 3a and b). Staining of xenograft tumor tissue with ki67, a specific nuclear marker for cell proliferation, showed that CRC tumor cells expressing IGPR-1 were strongly positive for ki67, indicating that IGPR-1 expressing tumors proliferated at a higher rate compared to tumor cells expressing empty vector (Figures 3c and f). In addition, the ectopic expression of IGPR-1 in mouse B16F melanoma cells also increased tumor growth both in cell culture and in mouse tumor xenograft assays (Supplementary Figures 4B, C).

**Figure 4:**
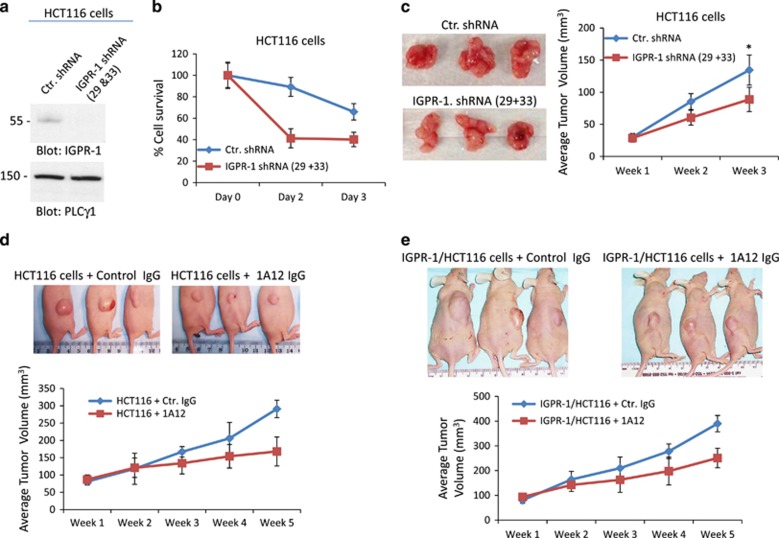
Knockdown of IGPR-1 by shRNA in HCT116 cells reduces tumor growth. HCT116 cells were transduced with a control lentivirus shRNA or two different IGPR-1 shRNAs (29 and 33). Whole-cell lysates were blotted with IGPR-1 or loading control protein, PLCγ1 (**a**). HCT116 cells expressing control shRNA or IGPR-1 shRNAs (29+33) were plated in non-adherent 24-well plates and viability of cells were measured by MTT assay (**b**). The same cell lines were xenografted into nude mice and growth of tumor cells were measured weekly for 21 days. The average tumor volume is shown. *P*<0.05 (**c**). HCT116 cells and HCT116 cells expressing IGPR-1 was xenografted into nude mice and after a one week inoculation, mice were intravenously injected with 10 μg control mouse IgG or 1A12 IGPR-1 blocking antibody. Tumor growth was monitored for up to 5 weeks and the average tumor growth is shown. *P*<0.05 (weeks 4 and 5) (**d**, **e**).

To demonstrate the endogenous function of IGPR-1, we knocked down IGPR-1 in HCT116 cells by shRNA and measured the survival of HCT116 cells in suspension. Despite the relatively low levels of IGPR-1 expression in HCT116 cells, depletion of IGPR-1 by shRNA markedly decreased survival of HCT116 cells in suspension (Figures 4a and b), and tumor growth in mouse (Figure 4c), underscoring the biological importance of IGPR-1 for tumor cell survival.

Having demonstrated the effect of IGPR-1 knockdown in the growth of HCT116 cells, we sought to explore the therapeutic targeting potential of IGPR-1 in CRC. To this end, we used a recently developed mouse monoclonal blocking antibody (1A12), which targets the extracellular domain of IGPR‐1 and inhibits phosphorylation of IGPR-1 on Ser220. Phosphorylation of IGPR-1 at Ser220 is critically required for IGPR-1 function to regulate angiogenesis.^30^ Treatment of HCT116 cells with 1A12 antibody reduced the survival of HCT116 cells in a dose-dependent manner (48%±5.6 inhibition at 10 μg/ml) compared to control IgG (Supplementary Figure 5A). The inhibitory effect of 1A12 in HCT116 cells was IGPR-1-dependent as knockdown of IGPR-1 in HCT116 cells obliterated the effect of 1A12 (Supplementary Figure 5B), whereas overexpression of IGPR-1 in HCT116 cells required a higher concentration of 1A12 antibody to inhibit growth of HCT116 cells in suspension (Supplementary Figure 5C). 1A12 antibody (10 μg/ml) reduced the survival of HCT116 cells by 48%, whereas at the same concentration of 1A12 antibody inhibited the survival of IGPR-1/HCT116 cells by 26% (Supplementary Figure 5A compared to 5C), indicating that overexpression of IGPR-1 in HCT116 cells increased tumor growth, which requires more blocking 1A12 antibody to reduce its pro-growth function.

Next, we examined whether 1A12 antibody could inhibit the *in vivo* growth of HCT116 cells. The results showed that weekly injection of 1A12 antibody (10 μg) inhibited the growth of HCT116 cells in mouse by 43% (average tumor volume for mice received control IgG was 291 mm^3^±25 vs mice receiving 1A12 IgG was 168 mm^3^±30) (Figure 4d). Similarly, 1A12 antibody (10 μg weekly injection) inhibited the growth of HCT116 cells expressing IGPR-1 by only 36% (average tumor volume for mice receiving control IgG was 390 mm^3^±33 vs mice receiving 1A12 IgG was 251 mm^3^± 39; Figure 4e). As noted, the anti-tumor efficacy of 1A12 antibody was significantly reduced by increasing expression of IGPR-1 in HCT116 cells, indicating that 1A12 antibody inhibits tumor growth by targeting IGPR-1. Taken together, the data demonstrate that IGPR-1 positively regulates CRC tumor growth and blocking its activity via 1A12 antibody inhibits tumor growth in mouse and in cell culture.

### IGPR-1 promotes multicellular aggregation to support tumor growth

To gain insight into the possible mechanism by which IGPR-1 promotes tumor cell growth, we posited that in the absence of attachment to substratum, tumor cells rely on IGPR-1for survival, which promotes multicellular aggregation. This mechanism, in turn, mitigates the adverse consequence of loss of adhesion and spreading that enables tumor cells to evade anoikis. To this end, we first asked whether multicellular aggregation provides a prosurvival signal for tumor cells. Accordingly, we seeded HCT116 cells in suspension with or without 0.5% methylcellulose and measured cell viability. Methycellulose inhibits cell–cell contact by creating a physical barrier between cells.^36, 37^ The data showed that the viability of HCT116 cells in suspension was significantly reduced in the presence of methylcellulose (Supplementary Figure 6A), demonstrating that multicellular aggregation increases tumor cell survival. Next, we examined whether IGPR-1 expressed in HCT116 cells contributes to multicellular aggregation. HCT116 cells ectopically expressing IGPR-1 displayed significantly larger cellular aggregates compared to HCT116 cells expressing empty vector (Supplementary Figure 6B). In addition, HCT116 cells expressing IGPR-1 incubated in suspension for 24 h formed significantly larger cellular aggregates (Supplementary Figure 6C). A similar effect was observed in HT29 cells expressing IGPR-1 (Supplementary Figure 6D). IGPR-1 undergoes trans-homophilic dimerization to regulate cell–cell adhesion.^30^ To demonstrate whether IGPR-1 directly contributes to formation of multicellular aggregates through trans-homophilic dimerization, we expressed the extracellular domain deleted IGPR-1 (ΔN-IGPR-1), which is unable to form trans-homophilic dimerization, in HT29 cells and examined their cellular aggregate formation. The result showed that ΔN-IGPR-1 was unable to stimulate multicellular aggregation (Supplementary Figure 6D).

**Figure 5:**
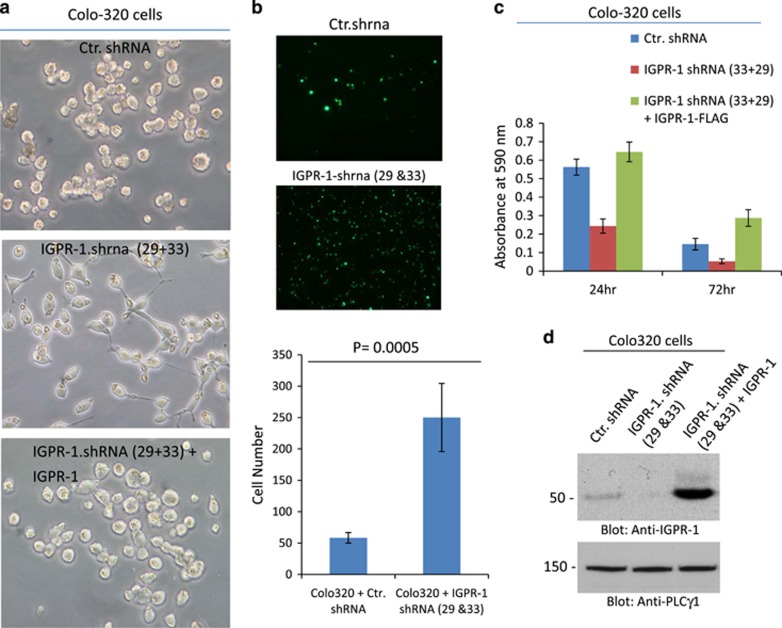
Knockdown of IGPR-1 by shRNA in Colo-320 adenocarcinoma cells decreases multicellular aggregation and increases cell spreading. Shown is morphology of Colo-320 cells expressing control shRNA, IGPR-1 shRNAs (29 & 33) or Colo-320 cells co-expressing IGPR-1 shRNAs (29 &33) and IGPR-1 (**a**). Colo-320 cells expressing control shRNA or IGPR-1 shRNAs (29 & 33) were plated on collagen-coated 24-well plates (50,000 cells/well) for 20 min. Cells were washed (2X) with PBS to remove the non-adhered cells. Cells were fixed, viewed under a fluorescence microscope and pictures were taken (**a**). Cells were counted from four randomly selected areas and average of adherent cells were presented (**b**). Colo-320 cells expressing control shRNA, IGPR-1 shRNAs (29+ 33) or co-expressing IGPR-1 shRNA with IGPR-1 were seeded in adherent 24-well plates in 1% FBS and viability of cells were measured after 24 and 72 h with MTT assay (**c**). Knockdown of IGPR-1 by shRNA in Colo-320 cells is shown (**d**).

To further examine the role of IGPR-1 in tumor multicellular aggregation, we knocked down IGPR-1 in the human colorectal adenocarcinoma cell line, Colo-320, and assessed the effect of loss of IGPR-1 on multicellular aggregation. We have chosen Colo-320 cells because these cells are uniquely loosely adherent and form multicellular aggregates in cell culture.^38^ The results showed that reducing expression of IGPR-1 in Colo-320 cells by shRNA profoundly altered their typical rounded and semi-adherent morphology (Figure 5a) and resulted in spreading and adhesion (Figure 5b). Re-expression of IGPR-1 reversed the observed effect of shRNA on the morphology of Colo-320 cells (Figure 5a), indicating that altered morphological changes in Colo-320 cells by IGPR-1 shRNA is directly associated with the loss of IGPR-1. Enhanced cell spreading of Colo-320 cells expressing IGPR-1 shRNA was further confirmed in a cell-spreading assay. Cells expressing IGPR-1 shRNA strongly adhered to collagen-coated plates compared to Colo-320 cells expressing control shRNA (Figure 5b). Next, we examined whether reduced multicellular aggregation of Colo-320 cells by IGPR-1 shRNA affected cell proliferation. The result showed that knockdown of IGPR-1 significantly reduced proliferation of Colo-320 cells and re-expression of IGPR-1 reversed this effect of IGPR-1 shRNA (Figure 5c). Taken together, the data demonstrate that tumor multicellular aggregation promotes cell survival and expression of IGPR-1 by tumor cells contributes to multicellular aggregation and tumor cell survival.

### IGPR-1 increases the resistance of CRC tumor cells to doxorubicin

**Figure 6:**
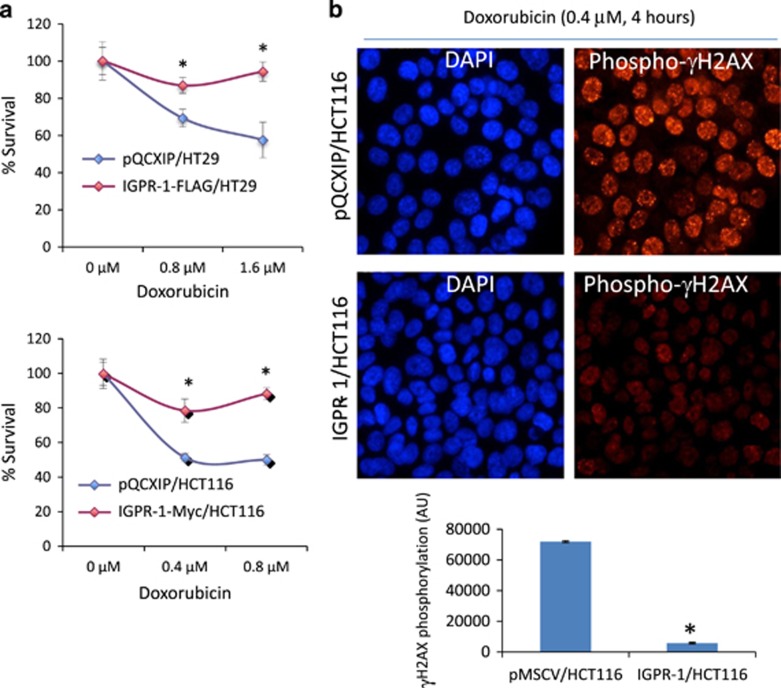
IGPR-1 increases the resistance of CRC tumor cells to doxorubicin. HT29 and HCT116 cells expressing empty vector (pMSCV) or IGPR-1 were seeded in non-adherent 24-well plates (5x10^4^/well) in 10% FBS in quadruple wells/group with increase dosage of doxorubicin. Cell viability was determined by MTT assay after 72 h treatment with doxorubicin (**a**). HCT116 cells expressing empty vector or IGPR-1 were treated with doxorubicin (0.4 μm, 4 h) and stained with phospho H2AX. The graph is quantification of phosphorylation of H2AX, representing two independent experiments (**b**).

Tumor cells often coopt to cellular aggregation by interacting with each other or other cell types to lessen the cytotoxic effects of chemotherapeutics, a phenomenon known as ‘multicellular resistance’.^39^ Multicellular resistance occurs in response to a variety of anti-cancer strategies, including chemotherapy and ionizing radiation.^12, 40^ Considering the effects of IGPR-1 on tumor multicellular aggregation, we examined whether IGPR-1expression in tumor cells influences the chemosensitivity of colon tumor cells to doxorubicin. We used doxorubicin because the mechanism of action of doxorubicin is well characterized.^41^ Expression of IGPR-1 in HT29 and HCT116 cells increased the resistance of tumor cells to the cytotoxic effect of doxorubicin (Figure 6a). To corroborate the observed effects of IGPR-1 on the chemosensitivity of colon tumor cells, we measured phosphorylation of histone H2AX which is called γH2AX, an early biomarker of the cellular DNA damage response (DDR) to chemotherapeutic agents.^42^ DDR functions as an anti-cancer response and is an attempt to suppress tumor cell growth by inducing cell death or replicative cellular senescence. DNA damage induced by chemotherapeutic agents results in rapid phosphorylation of histone H2AX at Ser139, which promotes cellular apoptosis.^43, 44^ The data demonstrated that HCT116 cells expressing IGPR-1 had significantly less γH2AX in response to doxorubicin treatment compared to control cells (Figure 6b), indicating that expression of IGPR-1 in tumor cells acts to delay or suppress the damage caused by doxorubicin. While, the mechanism by which IGPR-1 regulates phosphorylation of histone H2AX needs further investigation, the present data demonstrate that IGPR-1 by modulating phosphorylation of histone H2AX acts to reduce the sensitivity of tumor cells toward the DNA-damaging agent, doxorubicin.

### Doxorubicin induces phosphorylation of IGPR-1 at Ser220

**Figure 7:**
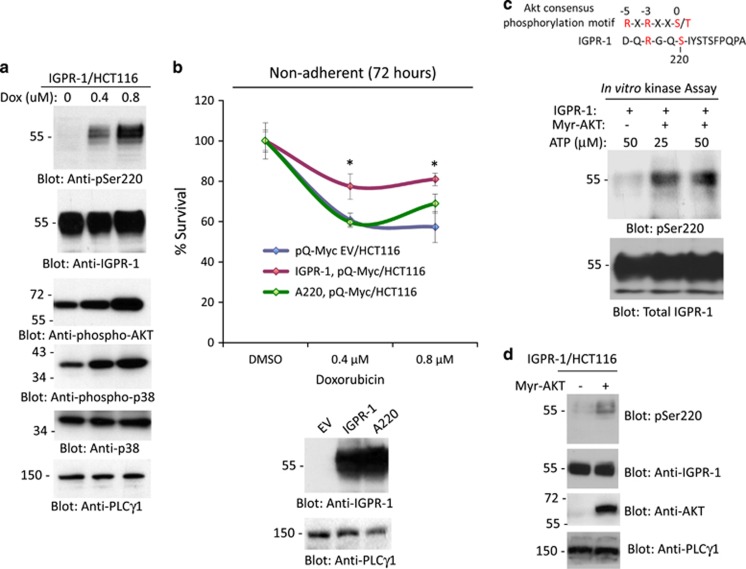
IGPR-1 is phosphorylated at Ser220 in response to doxorubicin and is required for IGPR-1-mediated resistance. HCT116 cells expressing IGPR-1 were treated with different concentrations of doxorubicin for 48 h. Cells were lysed and whole-cell lysates were blotted with anti-phospho-Ser220-IGPR-1, total IGPR-1, anti-phospho-AKT and anti-phospho-p38 antibodies. The same cell lysates were also blotted for PLCγ1 as a loading control (**a**). HCT116 cells expressing empty vector (pQ-Myc-EV), IGPR-1 or Ser220 mutant IGPR-1 (A220) were seeded in non-adherent 24-well plates and viability of cells were measured after 72 h (**b**). Expression of IGPR-1 and A220-IGPR-1 in HCT116 cells is shown (**b**). Immunoprecipitated myristoylated (Myr)-AKT was incubated with immunoprecipitated IGPR-1 with or without ATP and phosphorylation of IGPR-1 at Ser220 was detected by anti-phopsho-Ser220 antibody (**c**) and re-blotted for total IGPR-1 (**c**). The AKT consensus phosphorylation site is shown (**c**). Myr-AKT was co-expressed with IGPR-1 in HCT116 cells and whole-cell lysates were blotted for pSer220-IGPR-1, total IGPR-1, total AKT or for PLCγ1 for loading control (**d**).

In our recent study, we have demonstrated that IGPR-1 is phosphorylated at Ser220 through trans-homophilic dimerization and its phosphorylation is required for its adhesive function in endothelial cells.^30^ Therefore, we asked whether doxorubicin induces phosphorylation of Ser220 in HCT116 cells, which may contribute to the development of resistance. Curiously, doxorubicin treatment of HCT116 cells stimulated a robust phosphorylation of IGPR-1 at Ser220 in a dose-dependent manner (Figure 7a). Phosphorylation of Ser220 was detected after 24 h of treatment with doxorubicin and peaked significantly at 48 h (Supplementary Figure 7A). Previous studies have shown that doxorubicin stimulates activation of PI3 kinase/AKT and related protein kinases, ATM/ATR.^45, 46, 47^ In agreement with previous reports, we also observed an increase in the phosphorylation of AKT in HCT116 cells in response to doxorubicin (Figure 7a).

To examine the functional importance of phosphorylation of Ser220 in IGPR-1-mediated resistance of CRC tumor cells to doxorubicin, we ectopically expressed Ser220 mutant IGPR-1 (A220) in HCT116 cells and tested for their sensitivity to doxorubicin. The result showed that Ser220 mutant IGPR-1, unlike the wild-type IGPR-1, did not increase the resistance of HCT116 cells to doxorubicin (Figure 7b). Taken together, the data suggest that doxorubicin-induced phosphorylation of IGPR-1 at Ser220 determines the sensitivity of tumor cells to the killing effects of doxorubicin. Next, we decided to identify the possible kinases involved in the phosphorylation of Ser220. On the basis of the amino-acid sequence homology, Ser220 on IGPR-1was predicted to be a candidate substrate for AKT (Figure 7c). The conserved consensus AKT phosphorylation sequence is RXRXXS/T (where R, arginine; X, any amino acid; S, serine; T, threonine). The presence of arginine at the −3 position is particularly important for recognition by AKT, whereas arginine at the −5 position is considered less critical as there are several proteins that were identified as AKT substrates in which arginine at the −5 position was not conserved, including CREB (LSRRPS),^48^ SRPK2 (HDRSRT)^49^ and TTC3 (TPRSLS).^50^ To demonstrate whether Ser220 is phosphorylated by AKT, we performed an *in vitro* kinase assay using a purified constitutively active myristoylated AKT. The data demonstrated that AKT phosphorylates Ser220 on IGPR-1 (Figure 7c). Furthermore, overexpression of myristoylated AKT in HCT116 cells also increased phosphorylation of Ser220 (Figure 7d). Altogether, the data demonstrate that in response to the cytotoxic effects of doxorubicin, tumor cells trigger activation of a key prosurvival AKT pathway to phosphorylate IGPR-1 at Ser220, which in part significantly contributes to decreased sensitivity of tumor cells to the cytotoxic effects of doxorubicin.

Considering the observed importance of Ser220 phosphorylation on IGPR-1 function, we sought to examine whether Ser220 phosphorylation also contributes to IGPR-1-dependent tumor growth. First, we tested whether mutation of Ser220 inhibits the prosurvival function of IGPR-1 in HCT116 cells. The data showed that mutation of Ser220 significantly inhibited the prosurvival activity of IGPR-1 (Supplementary Figure 8A). Furthermore, the ability of Ser220 mutant IGPR-1 to stimulate growth of HCT116 cells in mouse tumor xenograft was also significantly reduced (Supplementary Figure 8B). Taken together, the data demonstrate a critical role for phosphorylation of Ser220 on IGPR-1function in tumor growth and the sensitivity of tumor cells to doxorubicin.

## Discussion

Much attention has focused on the role of tumor microenvironment in tumor growth and response to chemotherapeutics. This is particularly relevant to human colorectal cancer, in which, in addition to multiple genetic lesions, other cellular alterations also play major roles in the development of carcinoma.^51^ The work presented in this manuscript, for the first time, demonstrates that IGPR-1 expression is elevated in human primary colon cancers and promotes *in vivo* and *in vitro* tumor growth. Interfering with IGPR-1 activity by shRNA or blocking antibody inhibited growth of HCT116 cells, suggesting that targeting IGPR-1 could offer a novel anti-cancer strategy. IGPR-1 distinctively promotes tumor growth by increasing multicellular aggregation of tumor cells. More importantly, IGPR-1 expression in colon tumor cells significantly contributes to the development of resistance to the chemotherapeutic drug, doxorubicin. CRC is the second most common cause of cancer death in men and third in women.^52^ The response of CRC patients to current standard-of-care drugs is about 30–40%.^53^ Even with the advent of novel targeted anti-cancer drugs, not all patients respond to these agents, epitomizing the fundamental concept of personalized medicine in line with the heterogeneity of CRC. Therefore, further elucidation of IGPR-1 function in CRC could lead to the development of novel therapies that specifically target tumor cell aggregation.

CRC development is a multistep process characterized by numerous genetic and epigenetic lesions.^51, 54^ In addition to random genetic lesions, mechanisms by which CRC cells sense their microenvironment have significant importance in deriving the progression of malignancy and evasion from chemotherapy. As tumor cells lose the requirement for anchorage dependency to ECM for growth and survival, they increasingly rely on multicellular aggregation for survival. Multicellular aggregation is an evolutionary cellular property that is essential in the embryonic development of organisms. Tumor cells coopt to multicellular aggregation for their survival and, thereby, develop resistance to the killing effects of chemotherapeutics.^12, 39, 55, 56^ By forming multicellular aggregates, tumor cells function as ‘all for one and one for all’ in a self-defense mechanism that promotes survival and lessens their sensitivity to the cytotoxic effects of chemotherapeutics.^16, 57, 58^ Therefore, the multicellular aggregation of tumor cells represents a novel target for anti-cancer therapies and/or adjuvant treatments that aim to enhance CRC chemosensitivity. Our results demonstrate that IGPR-1 promotes tumor growth both *in vivo* and *in vitro* by increasing multicellular aggregation of tumor cells. Loss of adhesion is known to trigger activation of stress-induced pro-apoptotic p38 MAPK.^34, 35^ The data presented in this manuscript demonstrates that IGPR-1, by increasing multicellular aggregation suppresses activation of pro-apoptotic p38 activation.

The mechanisms by which tumor cells sense their microenvironment institute both protective and permissive roles in tumourigenesis.^59, 60^ Cell adhesion molecules (CAMs; cadherins, integrins and immunoglobulin superfamily molecules) permit tumor cells to sense their microenvironment, which enables them to resist apoptosis, sustain proliferation, invade and metastasize.^61, 62^ IGPR-1 appears to have no effect in the proliferation of cells in 2D cell culture condition, however, it promotes cell survival in non-adherent condition, suggesting that IGPR-1 could play a critical role in various stages of tumor metastasis, where tumor cells are not attached to their normal substratum. The acquisition of apoptotic resistance by tumor cells is one of the hallmarks of cancer,^62^ and, in many cases, the loss of functional p53 and expression of certain classes of CAMs help tumor cells to avoid cell death and survive.^63, 64, 65, 66^ In this regard, it is possible that increased IGPR-1 expression in tumor cells provides a distinct survival mechanism for these cells.

Another important aspect of IGPR-1 expression in colon cancer cells is its ability to modify the response of tumor cells to the chemotherapeutic effects of doxorubicin. IGPR-1 was uniquely phosphorylated at Ser200 in response to doxorubicin, in an AKT-dependent manner. Identification of Ser220 phosphorylation on IGPR-1 has significant implications for its use as a potential biomarker in response to treatment and tumor progression. Our data provide insights into the mechanisms by which tumor cells sense their microenvironment to impede apoptosis associated with the loss of adhesion to substratum and response to the killing effects of chemotherapeutics. We elucidated a molecular mechanism whereby IGPR-1 promotes tumor cell survival by multicellular aggregation. Furthermore, we have established the functional importance of IGPR-1 in CRC tumor cells in response to chemotherapeutics. IGPR-1 provides a unique ability to evade apoptosis, grow without anchoring to ECM, and develop resistance to conventional chemotherapies. Targeted therapies directed toward blocking tumor cells’ ability to sense their microenvironment, an unexplored mechanism that allows tumor cells to survive through multicellular aggregation, offers an attractive novel anti-cancer therapeutic approach.

## Materials and methods

### Chemicals, shRNAs and antibodies

Rabbit polyclonal anti-IGPR-1 antibody was made against a peptide derived from the cytoplasmic domain of IGPR-1^29^and anti-phospho-Ser220-IGPR-1 antibodies were previously described.^30^ Phospho-p38 antibody was purchased from Cell Signaling. Secondary HRP-conjugated rabbit and mouse antibodies were purchased from Santa Cruz. Human lentiviral IGPR-1 shRNAs cloned into pGIPZ vector (clone; V2LHS_19029), 3′-TGGTCTAGGAGAGACCCTG-5′ and clone (V2LHS_19033) 3′-TTTGCTGGCAGCTGCGGCG-5′ were purchased from Dharmacon (Lafayette, CO, USA).

### Plasmids and constructs

IGPR-1, ΔN-IGPR-1 and A220-IGPR-1 cDNA constructs were cloned into retroviral vector pMSCV.puro with c-Myc or FLAG tags. Chimeric IGPR-1 (cIGPR-1) was generated by PCR in which the extracellular domain of the human IGPR-1 was replaced with the human CSF-1 receptor (CSF-1R) and cloned into retroviral vector pMSCV.puro as described.^30^

### Cell culture, cell lines and virus production

HCT116 and HT29 cells (cells were confirmed by STR profiling by the source) were purchased from ATCC and maintained in RPMI supplemented with 10% fetal bovine serum (FBS) and penicillin/streptomycin. Retroviruses were produced in 293-GPG cells (cell line not authenticated). Viral supernatants were collected for 3 days and concentrated viruses were used to transduce into HT29 or HCT116 cells and infected cells were selected with puromycin. Lentiviruses shRNAs were produced in 293 T cells.

### Tumorsphere assay

HT29 and HCT116 cells expressing empty vector or IGPR-1 (5 × 10^4^/well, 400 μl of DMEM+1% FBS) were plated in 24-well low attachment plates (Corning) in quadruple wells per group. The 3-(4,5-dimethylthiazol-2-yl)-2,5-diphenyltetrazolium bromide (MTT) cell proliferation assay was performed as described by the manufacturer and read at an absorbance of 570 nm in a microplate reader (VERSA max, Molecular Devices, Sunnyvale, CA, USA). The resulting data were used to generate percent survival curves, with day 0 values serving as 100% survival baselines. All the MTT assays were repeated at least three times. In some experiments, cell survival in tumorsphere assays were subjected to flow cytometry analysis. In addition, in some experiments, cells were similarly prepared, but were treated with control vehicle or doxorubicin and cell viability was measured by MTT.

### Flow cytometry

Cells were trypsinized, washed twice with PBS and resuspended in Annexin V-binding buffer (Invitrogen, Carlsbad, CA, USA). Cells were then analyzed for phosphatidylserine exposure by staining with Annexin V APC (Biolegend), 7AAD (Biolegend, Dedham, MA, USA) and calcein violet (Ebioscience, San Diego, CA, USA). Briefly, samples were incubated with staining reagents for 20 min in the dark at room temperature, then placed on ice and immediately analyzed on BD LSR II SORP (Becton Dickinson) flow cytometer. A minimum of 50 000 events were collected in BD FACSDiva 6.2.1 (Becton Dickinson, Waltham, MA, USA) and then analyzed with FlowJo 10.0.6 software (FlowJo, Inc, Ashland, OR, USA). All data were acquired and analyzed at the Flow Cytometry Core Facility at BUSM.

### Mouse tumor xenograft assay

Female Nu/j mice (5-6 weeks old) were obtained from Jackson Laboratories. Each mouse was injected subcutaneously at the right flank with HCT116 or HT29 cells (5 × 10^6^) expressing empty vector or IGPR-1 mixed with growth factor-reduced and phenol red free Matrigel (Corning). Tumor size was measured in two dimensions using a caliper every week, and the tumor volume was expressed in mm3 using the formula: *V*=*0.5l* × *2w* (*l*, length and *w*, width) of the tumor. Tumor xenografts were grown for 21 days before animals were sacrificed and xenografts were removed, photographed and subjected to immunohistochemical analysis. In some experiments tumor cells were implanted for a week followed by weekly iv injection of control IgG or 1A12 antibody. Characterization of mouse monoclonal IGPR-1 blocking 1A12 antibody was recently described.^30^

### Immunohistochemistry (IHC)

For examination of xenograft tumor, tumors were fixed in 10% neutral buffered formalin, embedded in paraffin and sectioned. One slide (5 um section) per tumor was stained with hematoxylin and eosin (H&E) and another slide was stained for Ki67 (tumor proliferation marker) using a rabbit polyclonal anti-ki67 antibody (1:300 dilution, ab2960, EMD Millipore, Darmstadt, Germany). IHC staining was performed as per the manufacturer’s instruction using the EXPOSE Rabbit specific HRP/DAB detection IHC kit (Abcam, Cambridge, USA). At least three xenografts from each group were included in the analysis. Images were obtained using the iScan Coreo Au scanner (Ventana, AZ, USA).

### Statistical analyses

The Student’s two-tailed *t*-test (assuming equal variances) was used to analyze cell survival data in experiments comparing two cell lines. For experiments that compared three or more cell lines, the one-way analysis of variance with Tukey’s *post hoc* test was used to analyze the results. IGPR-1 levels were compared using non-parametric Mann–Whitney *U*-test. An alpha value of *P*<0.05 denoted a significant difference between two groups in all cell survival comparisons.

## Publisher’s note

Springer Nature remains neutral with regard to jurisdictional claims in published maps and institutional affiliations.
