# Supplementary material for: Targeting tumor multicellular aggregation through IGPR-1 inhibits colon cancer growth and improves chemotherapy
Source: Oncogenesis. 2017 Sep 18;6(9):e378–. doi: 10.1038/oncsis.2017.77 (PMC5623903; doi:10.1038/oncsis.2017.77)

Supplemental Data

IGPR-1 promotes multicellular aggregation to regulate colon cancer growth and response to chemotherapeutic

*Nicholas Woolf, *Bradley E Pearson, Philip A Bondzie, Rosana D. Meyer, Mehrdad Lavaei, Anna Belkina, Vipul Chitalia, Nader Rahimi

S. Figure 1. **Ectopic expression of IGPR-1 in HT29 and HCT116 cells does not affect cell viability in adherent cell culture condition**. HT29 and HCT116 cells expressing empty vector (pMSCV) or IGPR-1 were seeded in adherent 24-well plates ($5x10^4$/well) in low serum (1%FBS) in quadruple wells/group. Cell viability was determined by MTT assay at day 0, 2 and 4 (A, B). The graphs are representative of at least three independent experiments.

S. Figure 2: **Extracellular domain of IGPR-1 is required for its prosurvival activity**: Shown is expression of IGPR-1 and extracellular domain-deleted IGPR-1 (ΔN-IGPR-1) in HT29 cells (A). HT29 cells expressing IGPR-1 or ΔN-IGPR-1 were seeded in non-adherent 24-well plates (quadruple/group) and viability of cells were measured by MTT assay (B). Shown is schematic of chimeric IGPR-1 (cIGPR-1) and expression in HT29 cells (C). HT29 cells expressing cIGPR-1 were plated in adherent 24-well plates and after overnight serum starvation, cells were either left unstimulated or stimulated with CSF-1. Cell viability was measured by MTT assay.

S. Figure 3: Effect of inhibition of p38 in viability of HT29 cells. HT29 cells were seeded in non-adherent 24-well plates ($5x10^4$/well) in low serum (1%FBS) in quadruple wells/group with control vehicle or p38 kinase inhibitor, SB203580 (10µM). Cell viability was determined by MTT assay at day 0, 2 and 4 (A). HT29 cells expressing IGPR-1 were seeded in non-adherent 24-well plates ($5x10^4$/well) in low serum (1%FBS) in quadruple wells/group with control vehicle or SB203580 (10µM). Cell

viability was determined by MTT assay at day 4 (B). The graphs are representative of three independent experiments.

S. Figure 4: Expression of IGPR-1 in mouse melanoma B16F cells promotes tumor growth. Ectopic expression of IGPR-1 in B16F cells (A). B16F cells expressing empty vector or IGPR-1 were seeded in adherent 24-well plates ($5x10^4$/well) in low serum (1%FBS) in quadruple wells/group. Cell viability was determined by MTT assay at day 0, 2, 4 and 6 (B). B16F cells expressing empty vector or IGPR-1 were mixed with growth factor reduced matrigel and injected into mice (3 mice/group). Animals were sacrificed after 21 days, tumors were removed, picture was taken and tumors mass was measured (C).

S. Figure 5: Blocking IGPR-1 by a monoclonal blocking antibody inhibits growth of HCT116 cells. HCT116 cells were seeded in non-adherent 24-well plates ($5x10^4$/well) in 10%FBS in quadruple wells/group with increasing concentration of control mouse monoclonal antibody or IGPR-1 blocking monoclonal 1A12 antibody. Cell viability was determined by MTT assay at day 4 (A). Similarly, HCT116 cells expressing control shRNA or IGPR-1 shRNAs (29 & 33) were incubated with control IgG or 1A12 antibody and cell viability determined as Panel A (B). HCT116 cells expressing IGPR-1 was incubated with increasing concentrations of control IgG or 1A12 antibody and cell viability determined as Panel A. $^{*}P<0.05$.

S. Figure 6: Expression of IGPR-1 in HCT116 and HT29 cells promotes multicellular aggregation. HCT116 cells were seeded in non-adherent 24-well plates ($5x10^4$/well) in 10%FBS in quadruple wells/group with or without 0.5% methylcellulose (MC). Cell viability was determined by MTT assay at day 0, 2, and 4 (A). Equal numbers of HCT116 cells expressing empty vector or IGPR-1 were put on the shaker with a mild shaking for 15 minutes. Cells subsequently transferred to cell culture plates and

incubated for 5 minutes. Cell aggregates were viewed under microscope and pictures were taken. Cell aggregates were counted from three randomly selected fields and average number of cell aggregates is presented (B). The same cell groups were incubated in non-adherent 24-well plate for 48 hours and pictures were taken (C). HT29 cells expressing an empty vector, IGPR-1 or ΔN-IGPR-1 were subjected to aggregation assay as panel C (D).

S. Figure 7. Doxorubicin promotes phosphorylation of IGPR-1. HCT116 cells expressing empty vector (pQCXIP) or IGPR-1-Myc were treated with 0, 0.4, or 0.8μM doxorubicin (Dox) in suspension for 24 or 48 hours. Cells were lysed, and immunoblotted for phosphorylated IGPR-1 at serine 220 (pSer220) and total IGPR-1. The same cell lysates was also blotted for PLCγ1 as a loading control (A).

S. Figure 8: Phosphorylation of Ser220 is important for IGPR-1-mediated tumor growth in mouse. HCT116 cells ectopically expressing empty vector, wilt type IGPR-1 or Ser220 mutant IGPR-1 were mixed with growth factor-reduced matrigel and injected subdermally into mice (3 mice/group). After 21 days, mice were sacrificed, tumors were excised, pictures taken and tumor mass was measured (A, B).

Supplemental Data, Figure 1

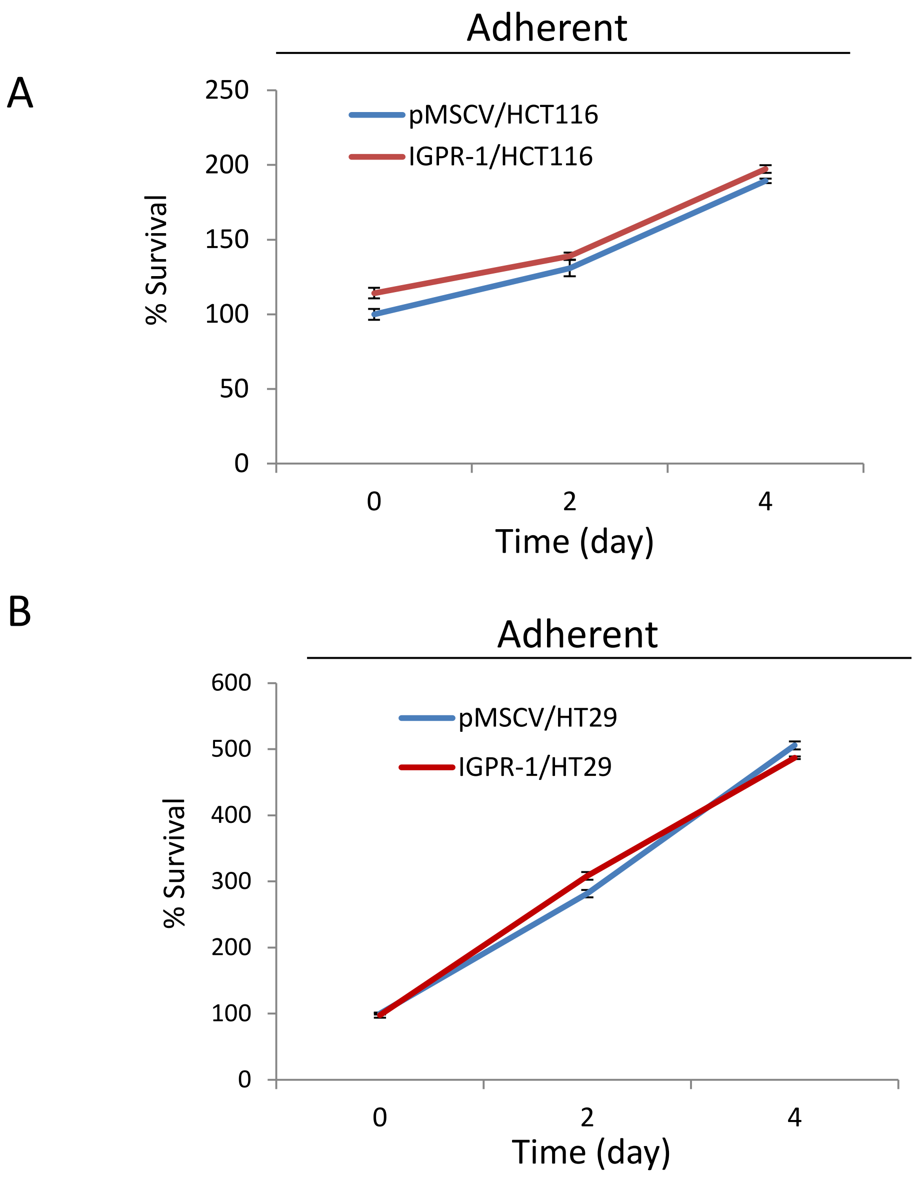

Supplemental Data. Figure 2

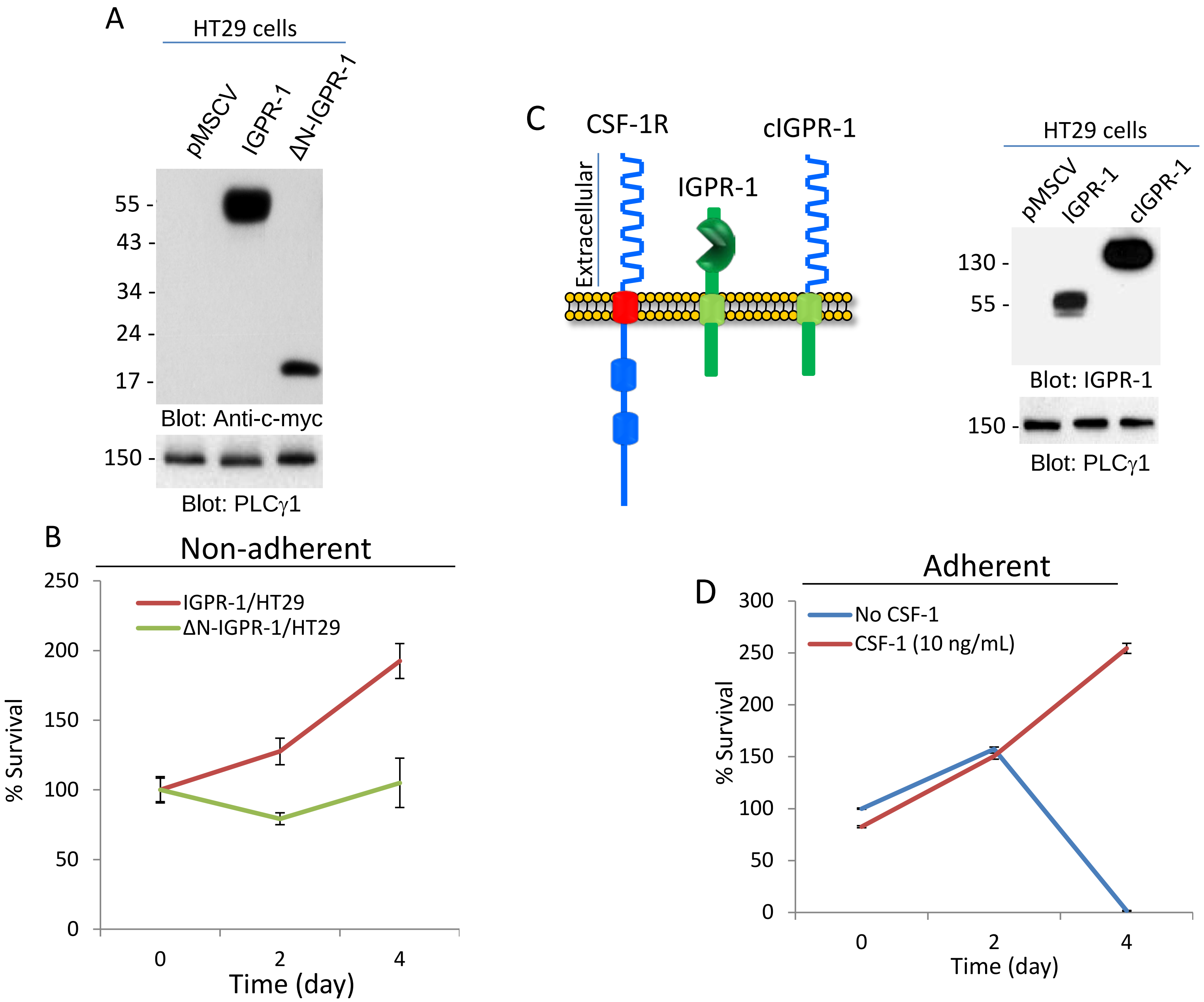

Supplemental Data, Figure 3

A

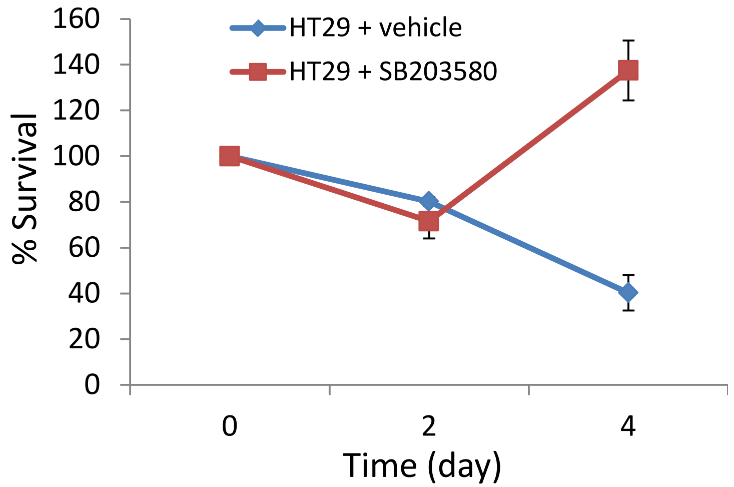

B

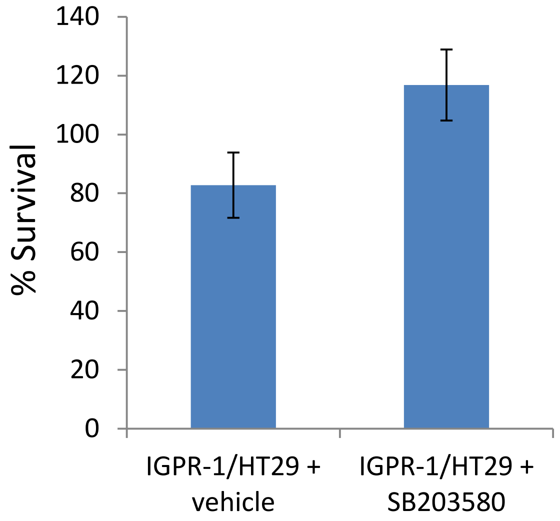

Supplemental Data, Figure 4

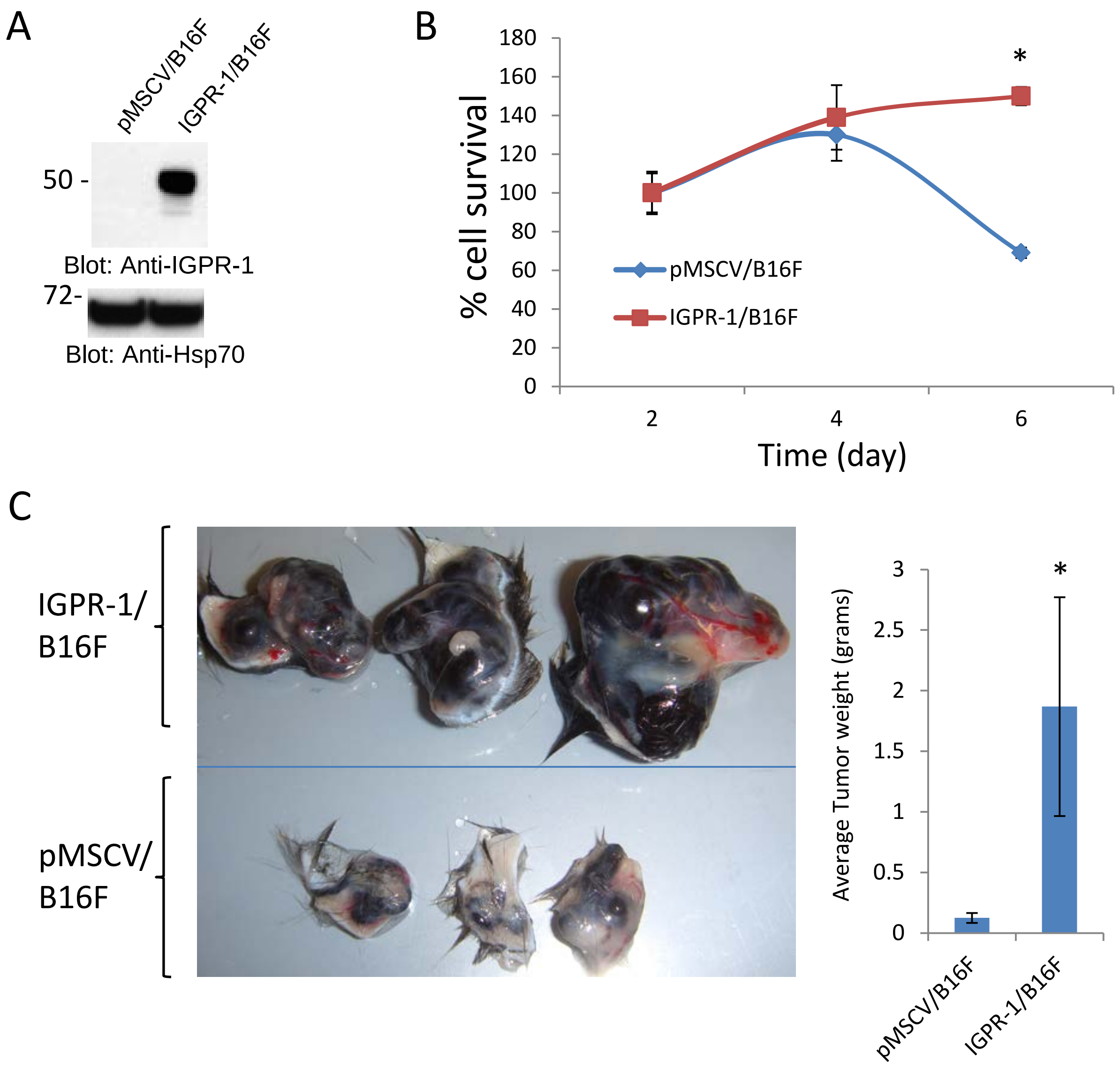

Supplemental Data, Figure 5

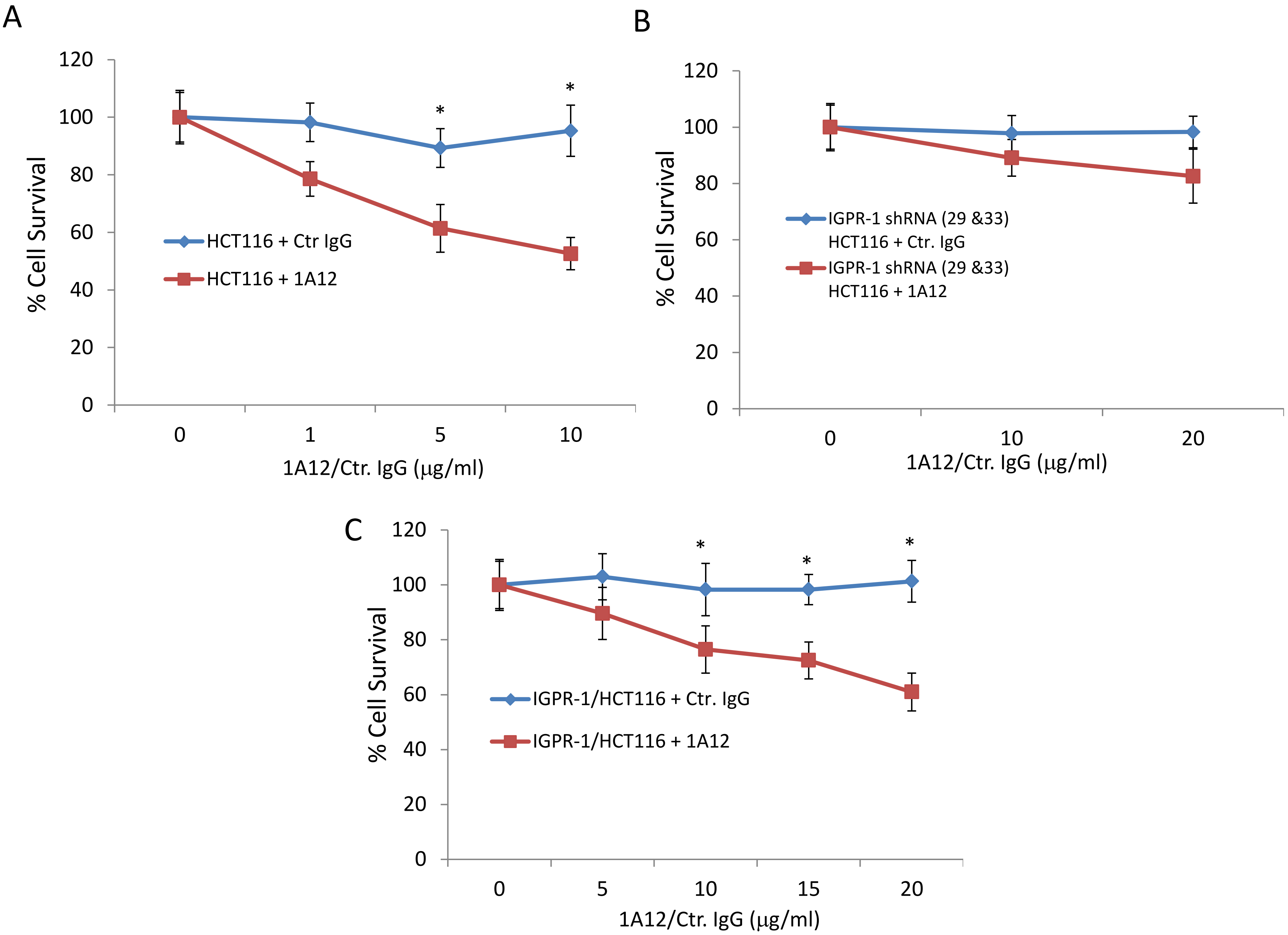

Supplemental Data, Figure 6

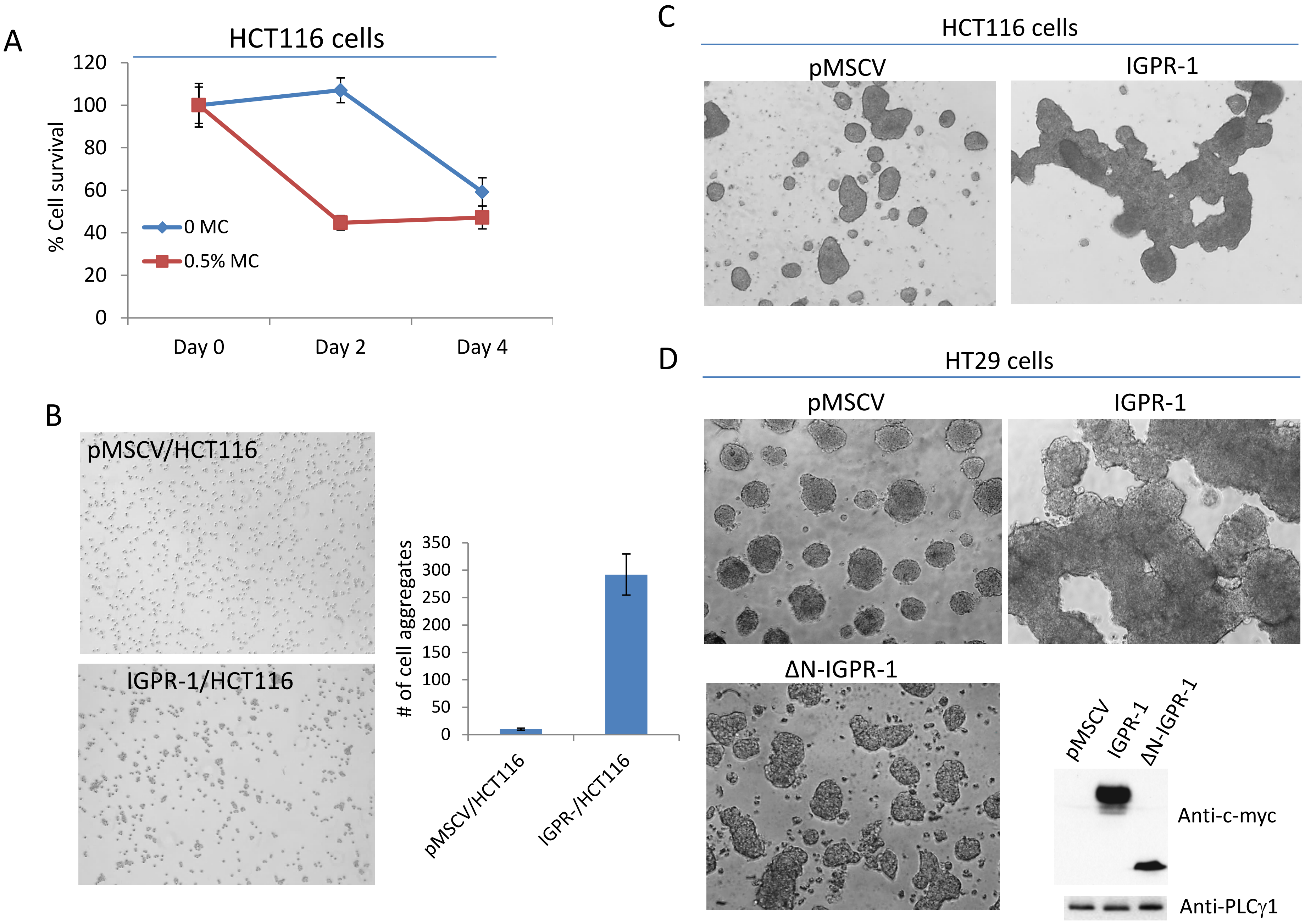

Supplemental Data, Figure 7

A

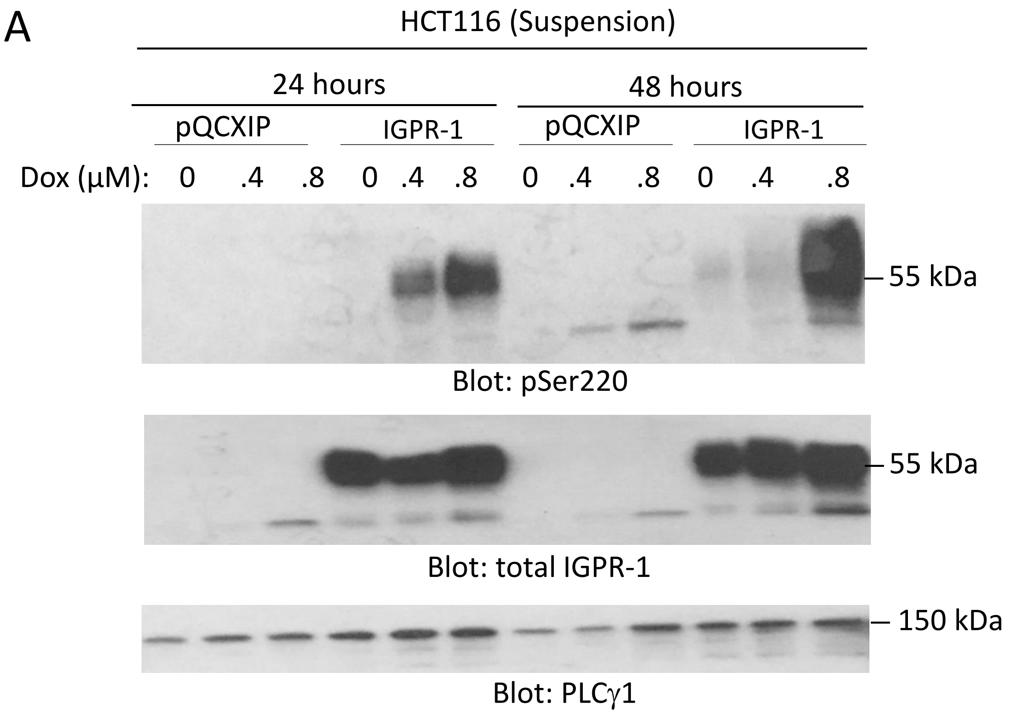

Supplemental Figure 8

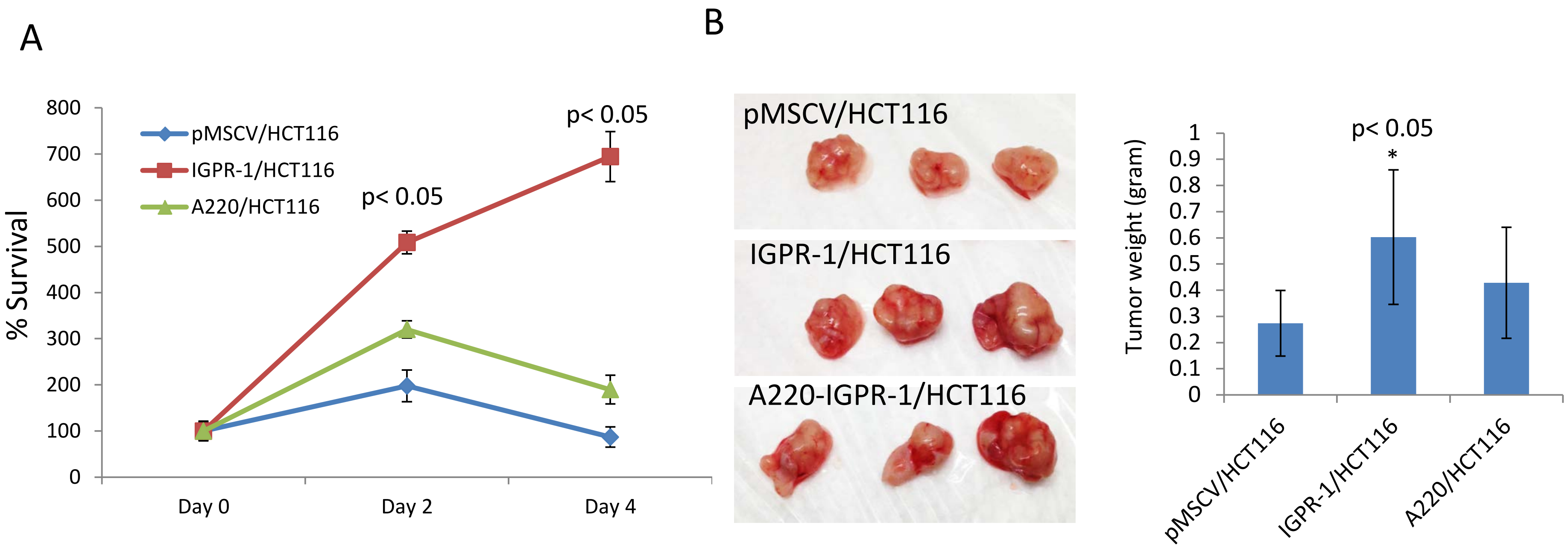

Supplement: Supplementary Information [file oncsis201777x1.pdf]
